# Utilizing point-of-care ultrasound in the cardiorenal clinic to enhance patient care

**DOI:** 10.1590/2175-8239-JBN-2020-0234

**Published:** 2021-01-29

**Authors:** Abhilash Koratala

**Affiliations:** 1 Medical College of Wisconsin, Milwaukee, USA.

In the recent past, there has been a growing interest in point-of-care ultrasonography (POCUS) in the field of nephrology, which was elegantly highlighted by Dr. Bastos and colleagues in their national survey^1^. Interestingly, 72% of the survey respondents had an ultrasound machine available for them to use at their institutions. While lack of training is a major barrier that has to be addressed over time, emphasizing the expanding role of POCUS in day-to-day practice outside the research environment hopefully motivates those nephrologists who have resources to adopt this skill.

Cardiorenal medicine is one such area where I would like to shed light on the utility of POCUS. It is well-recognized that lingering congestion is associated with worse outcomes in patients with cardiorenal syndromes. Unfortunately, conventional physical examination findings are not always reliable in assessing the volume status. In this context, POCUS has emerged as an attractive 'enhancement' to bedside clinical examination in the past two decades.

**Figure 1:**
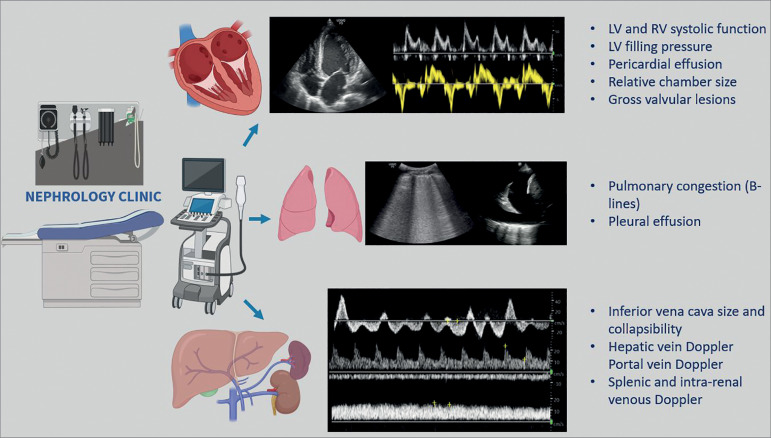
Various components of sonographic hemodynamic assessment performed in the outpatient cardiorenal clinic. Commonly posed focused clinical questions are listed. Representative sonographic images include - mitral inflow Doppler and mitral annular tissue Doppler (cardiac); B-lines and pleural effusion (pulmonary); Doppler waveforms of the hepatic, intra-renal and portal veins from top to bottom (venous). LV = left ventricle; RV = right ventricle.

At our institution, we have established an outpatient cardiorenal clinic, where comprehensive hemodynamic assessment using POCUS is performed by the nephrologist. We perform limited Doppler echocardiography, lung ultrasound, and Doppler assessment of the venous congestion in varying combinations to answer focused clinical questions (Figure 1) as well as longitudinally monitor the sonographic parameters. The findings are interpreted in conjunction with the overall clinical assessment and not in isolation. The rationale is to enhance patient care by leveraging the emerging data in support of POCUS-guided therapy to address congestion. For example, in a study of 1137 outpatients with chronic heart failure, patients whose management was guided by Doppler signs of elevated left ventricular filling pressure (n = 570) demonstrated a lower incidence of death (hazard ratio [HR] 0.45, 95%CI: 0.30-0.67, *p* < 0.0001), and death or worsening renal function (HR 0.49, 95%CI 0.36-0.67, *p* < 0.0001) compared to clinically-guided group^2^. Similarly, in a recent single-center randomized control trial, outpatients with heart failure managed with lung ultrasound-guided diuretic protocol had a significant decrease in the number of urgent visits for worsening heart failure (HR 0.518 for the primary end point, 95%CI 0.268-0.998, *p* = 0.049) as well as showed an improvement in the distance achieved in the 6-minute walking test^3^. Finally, quantifying venous congestion using the combined evaluation of inferior vena cava ultrasound, hepatic, portal, and intra-renal venous Doppler waveforms is a relatively novel concept showing promising results. For instance, in a cohort of 145 cardiac surgery patients, severe Doppler flow abnormalities in multiple veins with a dilated inferior vena cava (≥ 2 cm) has been associated with increased risk of acute kidney injury (HR: 3.69, 95%CI 1.65-8.24, *p* = 0.001)^4^. Moreover, these Doppler flow abnormalities improve with decongestive therapy allowing us to monitor the efficacy of treatment over a period of time^5^.

While the long-term outcomes of POCUS-guided therapy have to be established via larger clinical trials, ultrasonography is an age-old diagnostic tool with established safety profile and being able to utilize it to assess hemodynamic congestion at the bedside is a great addition to nephrologists' skill set.
